# Thoracic skeletal muscle mass predicts mortality in patients with surgery for pleural empyema: A case control study

**DOI:** 10.1111/1759-7714.15307

**Published:** 2024-04-10

**Authors:** Christian Galata, Philipp Schiller, Lukas Müller, Ioannis Karampinis, Davor Stamenovic, Roland Buhl, Michael Kreuter, Eric Dominic Roessner

**Affiliations:** ^1^ Department of Thoracic Surgery, Center for Thoracic Diseases, University Medical Center Mainz Johannes Gutenberg University Mainz Mainz Germany; ^2^ Department of Surgery, RoMed Hospital Rosenheim Rosenheim Germany; ^3^ Department of Radiology, University Medical Center Mainz Johannes Gutenberg University Mainz Mainz Germany; ^4^ Department for Pulmonology, Center for Thoracic Diseases, University Medical Center Mainz Johannes Gutenberg University Mainz Mainz Germany; ^5^ Center for Pulmonary Medicine, Department for Pulmonology, Center for Thoracic Diseases University Medical Center Mainz, Critical Care & Sleep Medicine, Marienhaus Clinic Mainz Mainz Germany

**Keywords:** complications, pleural empyema, prognosis, risk factor, thoracic skeletal muscle mass

## Abstract

**Background:**

This study investigated the role of the thoracic skeletal muscle mass as a marker of sarcopenia on postoperative mortality in pleural empyema.

**Methods:**

All consecutive patients (*n* = 103) undergoing surgery for pleural empyema in a single tertiary referral center between January 2020 and December 2022 were eligible for this study. Thoracic skeletal muscle mass index (TSMI) was determined from preoperative computed tomography scans. The impact of TSMI and other potential risk factors on postoperative in‐hospital mortality was retrospectively analyzed.

**Results:**

A total of 97 patients were included in this study. The in‐hospital mortality rate was 13.4%. In univariable analysis, low values for preoperative TSMI (*p* = 0.020), low preoperative levels of thrombocytes (*p* = 0.027) and total serum protein (*p* = 0.046) and higher preoperative American Society of Anesthesiologists (ASA) category (*p* = 0.007) were statistically significant risk factors for mortality. In multivariable analysis, only TSMI (*p* = 0.038, OR 0.933, 95% CI: 0.875–0.996) and low thrombocytes (*p* = 0.031, OR 0.944, 95% CI: 0.988–0.999) remained independent prognostic factors for mortality.

**Conclusions:**

TSMI was a significant prognostic risk factor for postoperative mortality in patients with pleural empyema. TSMI may be suitable for risk stratification in this disease with high morbidity and mortality, which may have further implications for the selection of the best treatment strategy.

## Graphical abstract

Outcomes and risk factors for in‐hospital mortality after surgery for pleural empyema. American Society of Anesthesiologists' (ASA) physical status classification; TSMI, thoracic skeletal muscle mass index.

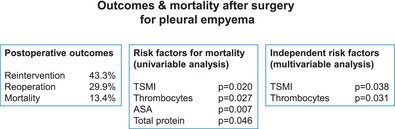

## INTRODUCTION

Pleural empyema is a serious condition which is associated with prolonged hospitalization and high healthcare costs. The incidence over the last decades is rising, particularly in the elderly and in cancer patients.^1^, ^2^ Therapeutic approaches include interventions like chest tube placement and the intrapleural application of fibrinolytic substances for early stages and thoracoscopic or open surgery for advanced stages.^3^, ^4^ Despite stage‐specific therapy, the mortality rate remains high and ranges between 10% and 20%.^5^ Therefore, research on risk factors associated with adverse outcomes in empyema patients is warranted.^6^, ^7^

One well‐established risk factor for adverse clinical outcomes in oncological and surgical patients is sarcopenia, defined as low skeletal muscle mass and strength by the European Working Group on Sarcopenia in Older People.^8^, ^9^ A good surrogate marker for sarcopenia is derived from imaging studies by determining the skeletal muscle mass index (SMI) by measuring the cross‐sectional area of skeletal muscles at the level of certain vertebral bodies. The SMI is known to correlate well with total muscle mass.^10^ Low SMI has been identified as a risk factor for poor outcomes in surgical patients in general as well as in patients with various lung diseases.^11^, ^12^, ^13^, ^14^

SMI has hardly been studied as a prognostic factor in patients with pleural empyema. This is noteworthy because patients with pleural empyema usually receive computed tomography (CT) scans, so SMI can be considered as a value‐added biomarker from opportunistic CT screening.^15^ Therefore, the aim of this study was to investigate the association of the thoracic skeletal muscle mass index (TSMI) with mortality in a cohort of surgically treated empyema patients.

## METHODS

### Patients

All consecutive patients undergoing surgery for pleural empyema between January 2020 and December 2022 at the Center for Thoracic Diseases, University Medical Center Mainz, Germany, were analyzed retrospectively. Of those, patients with CT scans of the chest available on the picture archiving and communication system at our institution in sufficient quality for thoracic muscle mass quantification were included in this study.

### Data acquisition

Data on patients and procedures were collected from medical records. Chest tube duration was defined as the time in days until removal of the last chest tube. Perioperative blood transfusion was defined as the transfusion of red blood cell concentrates from the time of surgery up to postoperative day 3. The thoracic morbidity and mortality classification system (TM&M) was used to grade surgical complications.^16^ Major morbidity was defined as complications ≥ grade 3. Mortality was recorded as in‐hospital mortality, defined as death occurring during the hospital stay after empyema surgery or death occurring after readmission within 30 days due to complications of empyema or empyema surgery.

### Thoracic skeletal muscle mass quantification

**FIGURE 1:**
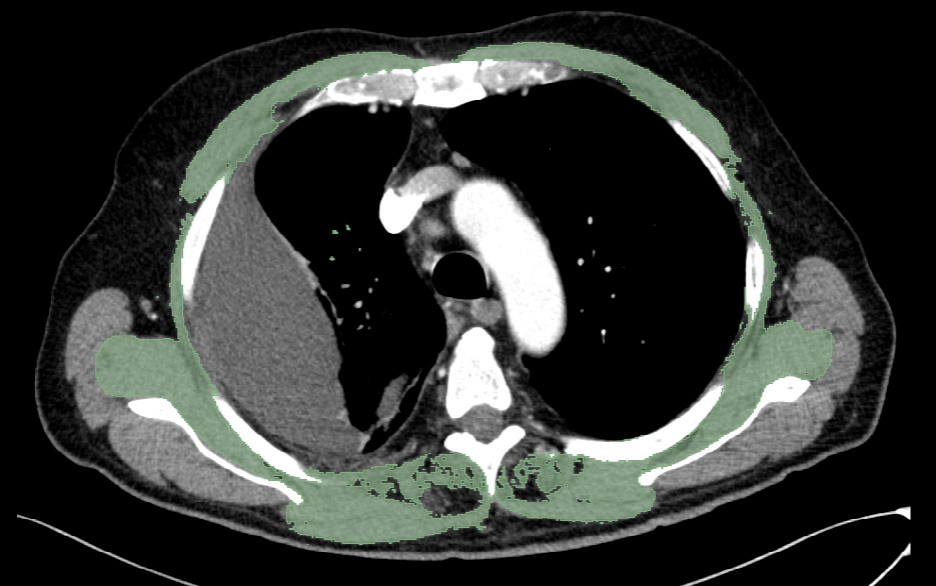
Sample axial chest CT image of the fourth thoracic vertebral region. Colored (green): cross sectional area of pectoralis, intercostalis, paraspinal, serratus, and latissimus muscles determined by semiautomatic muscle mass quantification. CT, computed tomography.

Contrast enhanced and unenhanced preoperative chest CT scans were used for thoracic skeletal muscle mass quantification. The procedure has been described and validated before.^13^, ^17^, ^18^ All chest CT scans were performed in a standardized position with the patient's arms positioned at the sides of the trunk following institutional protocols. All image analyses were performed by a radiologist specialized in thoracic imaging (L.M.). In brief, for each patient, measurements of the cross‐sectional area (CSA) of the pectoralis, intercostalis, paraspinal, serratus and latissimus muscles were performed at the fourth vertebral region (T4CSA). The cross‐sectional slides showing the middle of the fourth thoracic vertebra were selected by the investigator based on previous suggestions.^18^ Quantitative T4CSA assessment was then carried out semi‐automatically using a 3D slicer (version 5.2.2, Kitware Inc.) as shown in Figure 1.^19^ First, CSA was computed automatically by summation of the pixel attenuation of −30 to +150 Hounsfield units for skeletal muscle.^18^ Second, after applying the threshold method with a predefined Hounsfield unit threshold to slices, boundaries between different tissues were manually corrected. After determination of T4CSA for each patient, TSMI was calculated as T4CSA (in square centimeters) divided by body height in meters squared (in square centimeters per square meter).

### Statistical analysis

For quantitative variables, the median is presented with the interquartile range. Qualitative variables are shown as absolute numbers and relative frequencies. For bivariate analysis of categorical variables, *χ* ^2^ or Fisher's exact test was used, as appropriate. Bivariate analysis of continuous variables was carried out with independent samples student's *t*‐test. The Cochran‐Armitage test for trend was used to identify the association between a binary variable and an ordinal variable with >2 categories. A test result was considered statistically significant if *p* was less than 0.05. For the binary outcome “in‐hospital mortality” a multiple logistic regression analysis was performed. Variables were entered into the model if *p* was less than 0.05 in the univariable analysis. In the multiple analysis, the backward stepwise selection based on the probability of the Wald statistic was used and a significance level of *α* = 0.05 was chosen to determine final independent predictors. Odds ratios (OR) are presented together with their 95% confidence intervals (CI). All statistical tests were two‐tailed. The complete case approach was used for missing data. Analyses were performed using SPSS Statistics software (version 29.0, IBM).

## RESULTS

### Patient characteristics

Between January 2020 and December 2022, a total of 103 patients of both sexes underwent surgery for empyema at our department. In 97 of those cases, appropriate preoperative CT scans were available for analysis. A total of 97 patients were included in the study and patient characteristics are shown in Table 1. The median patient age was 68 years. Most patients were male (70.2%) and were categorized as American Society of Anesthesiologists' physical status class 3 (53.6%). A substantial number of the patients were active smokers at the time of empyema diagnosis (40.2%). History of malignant disease was seen in 27.8% of the cases, similar proportions had congestive heart failure (23.7%) and type 2 diabetes (24.7%). The presumed etiology of pleural empyema was parapneumonic in most cases (50.0%).

**TABLE 1:** Patient characteristics.

Variable	*n* / % / median	% / IQR
Male	70	72.2
Age (years)	68	60–79
BMI (kg/m^2^)	25.1	21.9–28.9
Current smoker	35	40.2
COPD	14	14.4
Malignant disease	27	27.8
Congestive heart failure	23	23.7
Dialysis	8	8.2
Diabetes	24	24.7
ASA		
1	1	1.0
2	8	8.2
3	52	53.6
4	34	35.1
5	2	2.1
Preoperative laboratory data		
Hb (13.5–17.5 g/dL)	9.8	9.1–11.6
Thrombocytes (150–360 /nL)	340	246–478
Leucocytes (x10^9^/L)	11.5	8.1–16.6
CRP (mg/L)	161	106–251
Creatinine (mg/dL)	0.8	0.6–1.1
INR	1.3	1.1–1.4
Total protein (g/L)	64	58–71
Bilirubin (mg/dL)	0.50	0.40–0.88
ASAT (U/L)	27	21–46
ALAT (U/L)	19	10–30
Empyema etiology		
Parapneumonic	48	50.0
Postoperative^a^	22	22.9
Other	26	27.1

Abbreviations: ALAT, alanine aminotransferase; ASA, American Society of Anesthesiologists; ASAT, aspartate aminotransferase; BMI, body mass index; COPD, chronic obstructive pulmonary disease; CRP, C‐reactive protein; Hb, hemoglobin; INR, international normalized ratio; IQR, interquartile range.

^a^ After previous thoracic, cardiac or abdominal surgery.

### Surgical procedures

Data on surgery are presented in Table 2. Minimally invasive surgery was feasible in 62.9% of the cases, while primary open surgery or conversion from video‐assisted thoracoscopic surgery to thoracotomy was needed in 37.1% of the patients. The most common surgical procedure was complete drainage and decortication of the lung (79.4%). A less radical approach with pleurolysis, drainage and partial decortication was used in 14.4% of the patients. The vast majority of patients were diagnosed intraoperatively to have stage 2 or stage 3 empyema (91.8%). The median length of surgery was 84 min, and the median length of chest tube duration and hospital stay were 6 and 13 days, respectively. Nearly one‐third of the patients (29.2%) required perioperative blood transfusions.

**TABLE 2:** Data on surgery.

Variable	*n* / % / median	% / IQR
Type of surgery		
VATS	61	62.9
Thoracotomy	36	37.1
Conversion (VATS to thoracotomy)	25	25.8
Surgical procedure		
Extended decortication	77	79.4
Pleurolysis and drainage	14	14.4
Thoracostomy	4	4.1
Lung resection	2	2.1
Empyema stage		
1	8	8.2
2	34	35.1
3	55	56.7
Surgical outcomes		
Length of surgery (min)	84	52–137
Length of stay (days)	13	7–21
Chest tube duration (days)	6	5–11
Pathogen detection	48	49.9
Blood transfusion	28	29.2
Morbidity and mortality		
Reoperation	29	29.9
Reintervention	42	43.3
Post anesthetic care unit	60	61.9
Intensive care (TM&M grade IV)	19	19.6
Hospital readmission	16	19.0
Mortality	13	13.4

Abbreviations: IQR, interquartile range; TM&M, thoracic morbidity and mortality score; VATS, video‐assisted thoracoscopic surgery.

### Morbidity and mortality

Two‐thirds of the patients (61.9%) were monitored on high dependency or intensive care units postoperatively. Intensive care unit management for potentially life‐threatening conditions was required in 19.6% of the cases. After surgery, nearly half of the patients underwent reintervention (43.3%) and surgical revision was performed in 29.9% of the cases. The indication for reintervention or surgical revision depended on the clinical condition of the patient and included cases with postoperative pleural effusion, empyema recurrence or postoperative abscess or hemothorax. The in‐hospital mortality rate was 13.4%. Of the 84 patients who were able to be discharged from the hospital, 19.0% were readmitted with postoperative complications.

### Risk factors for in‐hospital mortality

Several potential clinical risk factors were investigated with regard to postoperative mortality. Table 3 lists these parameters together with the results of univariable and multivariable analyses. In univariable analyses, low values for preoperative TSMI (*p* = 0.020), low preoperative thrombocyte levels (*p* = 0.027), low preoperative levels of total serum protein (*p* = 0.046) and higher preoperative ASA category (*p* = 0.007) were statistically significant risk factors for postoperative mortality. When multivariable analysis was performed, only TSMI (*p* = 0.038, OR 0.933, 95% CI: 0.875–0.996) and thrombocytes (*p* = 0.031, OR 0.944, 95% CI: 0.988–0.999) remained in the statistical model as independent prognostic factors for postoperative mortality.

**TABLE 3:** Factors for in‐hospital mortality in univariable and multivariable analysis.

Variable	Univariable	Multivariable	
	*p*‐value	*p*‐value	Odds ratio (95% CI)
TSMI	0.020*	0.038*	0.933 (0.875–0.996)
Thrombocytes	0.027*	0.031*	0.944 (0.988–0.999)
ASA	0.007*	0.224	
Total protein	0.046*	0.099	
Age	0.070		
BMI	0.916		
Gender	1.000		
COPD	0.091		
Current smoker	0.750		
Malignant disease	0.506		
Chronic heart failure	0.290		
Diabetes	1.000		
Dialysis	0.072		
CRP	0.379		
Leukocytes	0.727		
Creatinine	0.318		
Bilirubin	0.994		
ASAT	0.090		
ALAT	0.282		
INR	0.383		
Hb	0.475		
Empyema stage	0.214		
Empyema etiology	0.935		
Type of surgery (VATS vs. open)	0.066		
Length of surgery	0.205		
Surgical procedure	0.173		
Pathogen detection	0.553		
Blood transfusion	0.190		
Reintervention	0.069		
Reoperation	1.000		

*Note*: Variables with statistical significance in univariable analysis (T4MI, thrombocytes, ASA classification, total protein) were entered into the multivariable model. In the multivariable analysis, only T4MI and thrombocytes remained in the model as independent risk factors. Asterisks (*) indicate statistical significance.

Abbreviations: ALAT, alanine aminotransferase; ASA, American Society of Anesthesiologists' physical status classification; ASAT, aspartate aminotransferase; BMI, body mass index; CI, confidence interval; COPD, chronic obstructive pulmonary disease; CRP, C‐reactive protein; Hb, hemoglobin; INR, international normalized ratio; IQR, interquartile range; TSMI, skeletal muscle mass index at the fourth thoracic vertebra; VATS, video‐assisted thoracoscopic surgery.

## DISCUSSION

To the best of our knowledge, this is the first study to evaluate skeletal muscle mass as a prognostic factor in patients with pleural empyema. The main finding was that a low TSMI was an independent risk factor for mortality in a cohort of 97 consecutive surgical patients. With the widespread availability of chest CT scans in empyema patients, TSMI may be useful for risk stratification in this disease with persistently high morbidity and mortality. Given the rapid adoption of automated image analysis tools, TSMI can easily and quickly be determined in clinical practice, turning it into a value‐added biomarker from opportunistic CT screening.^15^

Further studies are needed to evaluate whether TSMI can be used to select empyema patients that may benefit from upfront surgery versus those in whom a step‐up approach with preoperative patient optimization followed by surgery is advisable.

Our data confirm that pleural empyema is a disease associated with high morbidity and mortality.

The postoperative reintervention rate was 43.3%, and 19.6% of the patients required therapy on an intensive care unit (TM&M Grade IV). The in‐hospital mortality rate was 13%. These findings are consistent with previously published data of patients with pleural space infections, which reported mortality rates ranging from 10% to 20%.^5^, ^6^, ^20^ A recent, large retrospective analysis by Bobbio et al. observed a mortality rate of 17.1% in a large cohort of 25 512 hospitalizations for pleural empyema.^6^

The baseline characteristics of the patients in our cohort are comparable with the literature. As in previous analyses, we investigated an elderly cohort (median age 68 years) with a male predominance (72.2%) and parapneumonic (50.0%) and postoperative (23.9%) empyema as the most common etiologies.^1^, ^6^ Regarding the surgical approach, previous results are very variable with conversion rates (minimally invasive to open surgery) of up to 61%.^21^, ^22^, ^23^ We observed a rate of 37.1% of primary open or converted to open procedures.

Several previous studies have identified factors associated with poor outcomes in empyema patients. The recent, large French national database analysis by Bobbio et al. found lung resection and diagnosis of cancer as risk factors for mortality. However, sarcopenia or SMI were not investigated in this study.^6^ Based on data from previous retrospective analyses and on the MIST1 and 2 trials, Rahman et al. developed a risk score (RAPID) containing the parameters urea, age, fluid purulence, source of infection and diet.^4^, ^20^, ^24^ This score has been validated by the same research group; however, prospective data on the use of the RAPID score in the management of patients with empyema are not yet available.^25^

What our study adds is that TSMI may be an independent predictor of mortality in empyema patients. As mortality is closely linked to complications, low TSMI might also be useful in anticipating adverse events in these patients, although this cannot be concluded from the results of our present study. The relevance of TSMI for clinical outcomes in pleural empyema does not seem far‐fetched. Previously, low SMI as a surrogate marker for sarcopenia has been linked to reduced overall survival in oncology patients and to an increased risk of complications in surgery.^11^, ^26^, ^27^

The central goal of research on clinical risk factors is to identify those parameters that can be modified to positively impact patient outcomes. The recognition of sarcopenia as a key player this regard has contributed to the widespread adoption of preoperative patient optimization protocols, for example, in the treatment of inflammatory bowel diseases, and encouraged the roll‐out of structured prehabilitation programs in elective surgery and oncology alike. Such programs have been shown to reduce complications and improve survival.^28^, ^29^ As an example, in surgery for Crohn's disease, it is good clinical practice to identify patients with bad nutritional status and sarcopenia and, if possible, postpone surgery in favor of preoperative patient optimization including antibiotics, interventional drainage and enteral and parenteral nutritional therapy.^30^ Similarly, TSMI evaluation could contribute to risk stratification in empyema patients planned for decortication and help to separate patients suitable for upfront surgery from high‐risk patients where bridging to surgery with antibiotics, interventional drainage and nutritional therapy might be advisable. This approach is also supported by one of the few current guidelines on the subject.^31^

Furthermore, in case of surgery, TSMI could also guide the decision‐making process on the optimal surgical approach, for example, primary (open) complete decortication versus primary minimally invasive partial decortication. These questions are particularly interesting as there is still an ongoing debate whether empyema patients benefit from early surgical intervention as a first‐line strategy or whether surgery should follow if conservative management fails.^32^, ^33^, ^34^

There were several limitations to this study. It was a single center retrospective analysis, which carries an inherent risk of bias and limits the validity of the findings. There may be unknown factors that were not assessed. The number of patients did not allow the analysis of all subgroups of interest. Moreover, the number of patients with mortality was low, which limits the statistical significance of the results. Regarding SMI, in most studies, muscle mass was measured at the level of the third lumbar vertebral body (L3). As abdominal imaging including L3 is often not available in thoracic patients, we determined TSMI measuring muscle mass at the level of the fourth thoracic vertebral body, as previously described.^13^, ^35^ However, TSMI is not only a surrogate marker for sarcopenia, but also indicates the muscle mass available for respiration. Therefore, beyond the adverse effects of sarcopenia in general, low TSMI may have a direct impact on pulmonary function and increase disease severity.^36^

To the best of our knowledge, this is the first analysis of thoracic skeletal muscle mass as a prognostic factor in patients with pleural empyema. TSMI was a significant risk factor for mortality in the overall cohort. Due to the wide availability of preoperative CT scans and automated image analysis tools, TSMI as a value‐added opportunistic biomarker may be suitable for risk stratification in patients with pleural empyema. This may have further implications for the selection of the best treatment strategy for a patient group with relevant mortality.

## AUTHOR CONTRIBUTIONS

All authors had full access to the data in the study and take responsibility for the integrity of the data and the accuracy of the data analysis. Christian Galata: Conceptualization, methodology, validation, formal analysis, investigation, data curation, writing—original draft and visualization. Philipp Schiller: Conceptualization, methodology, validation, formal analysis, investigation, data curation, writing—original draft and visualization. Lukas Müller: Conceptualization, methodology, software, validation, investigation, writing—review and editing and visualization. Ioannis Karampinis: Conceptualization, methodology, validation, writing—review and editing. Davor Stamenovic: Conceptualization, methodology, validation, writing—review and editing. Roland Buhl: Conceptualization, resources, writing—review and editing, supervision and project administration. Michael Kreuter: Conceptualization, resources, writing—review and editing, supervision and project administration. Eric Dominic Roessner: Conceptualization, resources, writing—review and editing, supervision and project administration.

## CONFLICT OF INTEREST STATEMENT

The authors report no conflict of interest.
